# Bifidobacteria Strain Typing by Fourier Transform Infrared Spectroscopy

**DOI:** 10.3389/fmicb.2021.692975

**Published:** 2021-09-13

**Authors:** Francesca Deidda, Nicole Bozzi Cionci, Miriam Cordovana, Ilenia Campedelli, Fabio Fracchetti, Diana Di Gioia, Simone Ambretti, Marco Pane

**Affiliations:** ^1^Probiotical Research S.r.L., Novara, Italy; ^2^Department of Agricultural and Food Sciences, University of Bologna, Bologna, Italy; ^3^Bruker Daltonik GmbH, Bremen, Germany; ^4^Microbion S.r.L., San Giovanni Lupatoto, Verona, Italy; ^5^Microbiology Unit-University Hospital of Bologna Policlinico Sant’Orsola-Malpighi, Bologna, Italy

**Keywords:** *Bifidobacterium*, probiotics, FTIR spectroscopy, strain typing, PFGE, MLST, live biotherapeutic products

## Abstract

Fourier transform infrared (FTIR) spectroscopy, a technology traditionally used in chemistry to determine the molecular composition of a wide range of sample types, has gained growing interest in microbial typing. It is based on the different vibrational modes of the covalent bonds between atoms of a given sample, as bacterial cells, induced by the absorption of infrared radiation. This technique has been largely used for the study of pathogenic species, especially in the clinical field, and has been proposed also for the typing at different subspecies levels. The high throughput, speed, low cost, and simplicity make FTIR spectroscopy an attractive technique also for industrial applications, in particular, for probiotics. The aim of this study was to compare FTIR spectroscopy with established genotyping methods, pulsed-field gel electrophoresis (PFGE), whole-genome sequencing (WGS), and multilocus sequence typing (MLST), in order to highlight the FTIR spectroscopy potential discriminatory power at strain level. Our study focused on bifidobacteria, an important group of intestinal commensals generally recognized as probiotics. For their properties in promoting and maintaining health, bifidobacteria are largely marketed by the pharmaceutical, food, and dairy industries. Strains belonging to *Bifidobacterium longum* subsp. *longum* and *Bifidobacterium animalis* subsp. *lactis* were taken into consideration together with some additional type strains. For *B. longum* subsp. *longum*, it was possible to discriminate the strains with all the methods used. Although two isolates were shown to be strictly phylogenetically related, constituting a unique cluster, based on PFGE, WGS, and MLST, no clustering was observed with FTIR. For *B. animalis* subsp. *lactis* group, PFGE, WGS, and MLST were non-discriminatory, and only one strain was easily distinguished. On the other hand, FTIR discriminated all the isolates one by one, and no clustering was observed. According to these results, FTIR analysis is not only equivalent to PFGE, WGS, and MLST, but also for some strains, in particular, for *B. animalis* subsp. *lactis* group, more informative, being able to differentiate strains not discernible with the other two methods based on phenotypic variations likely deriving from certain genetic changes. Fourier transform infrared spectroscopy has highlighted the possibility of using the cell surface as a kind of barcode making tracing strains possible, representing an important aspect in probiotic applications. Furthermore, this work constitutes the first investigation on bifidobacterial strain typing using FTIR spectroscopy.

## Introduction

Fourier transform infrared (FTIR) spectroscopy is a technology traditionally used in chemistry to determine the molecular composition of a wide range of sample types, and it is based on the different vibrational modes of covalent bonds induced by the absorption of infrared (IR) radiation (Griffiths and De Haseh, 2007; Berthomieu and Hienerwadel, 2009). Starting from the 1990s, this methodology has been largely applied to the microbiology field for the discrimination, classification, and identification of microorganisms at different taxonomic levels, such as genera, species, and even strain level (Helm et al., 1991; Naumann et al., 1991; Novais et al., 2019). The power of FTIR consists of producing an IR spectrum composed of many different vibrational modes of all cellular components, allowing the discrimination of microbial cells in a non-destructive manner (Davis and Mauer, 2010). Each bacterial cell exhibits a unique FTIR spectrum, corresponding to its specific fingerprint signature and correlating with genetic information (Helm et al., 1991; Naumann et al., 1991; Lasch and Naumann, 2015).

Several studies using FTIR spectroscopy focused on foodborne, clinical, and epidemiological pathogens, e.g., *Escherichia coli*, *Salmonella enterica*, *Streptococcus pneumoniae*, and *Listeria monocytogenes* (Preisner et al., 2010; Fetsch et al., 2014; Nyarko et al., 2014; Novais et al., 2019), and have recently paved the way for outbreak investigation (Martak et al., 2019; Hu et al., 2020). Particularly, as FTIR was broadly explored for bacterial classification at the species level, it was also proposed for bacterial typing at the subspecies level, aiming to find an alternative to the established, but very expensive, time-consuming and not applicable on large-scale DNA-based techniques and other methods with high discriminatory power commonly used for epidemiological purposes. Among these, multilocus sequence typing (MLST) (Maiden et al., 1998; Enright and Spratt, 1999) and pulsed-field gel electrophoresis (PFGE) have been largely used for outbreak monitoring and examination (Neoh et al., 2019). In this context, whole-genome sequencing (WGS), which can provide consistent genetic information, has become the new gold standard for identifying, comparing, and classifying microorganisms (Gilchrist et al., 2015). However, besides the discriminatory power, it is necessary to consider the high-cost, laborious, and time-consuming laboratory work related to these technologies, which usually limits their routine application (Sabat et al., 2013).

Fourier transform infrared has been already successfully investigated for subtyping *Yersinia enterocolitica*, *Staphylococcus aureus*, *Klebsiella pneumoniae*, *L. monocytogenes*, and *E. coli* (Quintelas et al., 2018) and recently started to be implemented into the laboratory routine (Novais et al., 2019). The main advantages related to this method are the analysis time, cost, laboratorial simplicity, absence of chemical reagents, and very low sample amount per analysis, in addition to relevant information about the biomolecular content of the microorganisms including lipids, carbohydrates, proteins, and nucleic acids deriving from the IR spectrum (Quintelas et al., 2018). In 2018, Bruker commercialized an automated typing system based on FTIR technology, the IR Biotyper (Bruker GmbH, Bremen, Germany), whose application has gained large interest, becoming common in typing bacterial isolates especially in the field of clinical microbiology (Bruker GmbH Daltonics Division, 2018; Burckhardt et al., 2019; Martak et al., 2019; Hu et al., 2020).

All these features contribute to make the FTIR a fascinating and attractive technique not only for clinical and epidemiological investigation but also for other sectors related to the microbiology field, such as the probiotic industry. In the last 20 years, the attention for probiotic microorganisms has increased in both researchers and consumers, promoting the maintenance of health status and host wellness. Distinguishing probiotic products is challenging due to differences in their mechanisms of action, manufacturing processes, quality control, and efficacy of different strains. The production of good-quality probiotics is already fundamental in the early stages of the process, and it is not limited to the only biomass growth. As many properties can affect the development of probiotics, in order to guarantee quality, stability, and safety of the product, strain-specific verification is required. Moreover, in the clinical field, the efficacy of probiotics has been demonstrated to be clearly strain- and disease-specific (McFarland et al., 2018); therefore, the choice of the appropriate strain for the patient can be challenging. In these contexts, FTIR can constitute a quick and reliable technique for typing probiotic bacteria and an efficient tool for identifying a target probiotic product.

Bifidobacteria are considered key commensals in human–microbe interactions and are recognized to play an important role in maintaining a healthy gut (Turroni et al., 2017). These health-promoting bacteria are widely used as probiotics in preventive and therapeutic strategies for human diseases, especially in pediatric subjects, for their capability of reaching and colonizing the gastrointestinal tract, their long history of safe use, and their documented health benefits (Biavati et al., 2000; Leahy et al., 2005; Sanders et al., 2010; Di Gioia et al., 2014; Bozzi Cionci et al., 2018).

This study was aimed to test the potential discriminatory power of FTIR technology at strain level among members of the *Bifidobacterium* genus recognized as probiotics in order to pave the way for the introduction of this new phenotyping-type method into routine process for the development of probiotic products. Specifically, the FTIR performance was compared with other assessed genotyping techniques, PFGE, WGS, and MLST, with a particular focus on the strains belonging to *Bifidobacterium longum* subsp. *longum* and *Bifidobacterium animalis* subsp. *lactis*.

## Materials and Methods

### Bacterial Strains and Growth Conditions

A total of four *B. longum* subsp. *longum* and four *B. animalis* subsp. *lactis* strains commonly used as probiotics were included in this study, and the type strains *B. longum* subsp. *longum* DSM20219^T^ and *B. animalis* subsp. *lactis* DSM10140^T^ were used as reference controls (Table 1). Further four species belonging to *Bifidobacterium* were included in the PFGE and FTIR analysis to evaluate the discriminatory power of the FTIR method at species level (Table 2).

**TABLE 1:** Strains included in the PFGE, FTIR, WGS, and MLST analyses. *Bifidobacterium longum* subsp. *longum* BL-CT and *Bifidobacterium animalis* subsp. *lactis* BS-CT are type strains.

**Species**	**Code**	**Deposit code**	**GenBank accession number**	**Techniques used**
*B. longum* subsp. *longum*	BL03	DSM16603	JAGGDB000000000	PFGE, FTIR, WGS, and MLST
*B. longum* subsp. *longum*	W11	LMG P-21586	MRBG00000000.1	PFGE, FTIR, WGS, and MLST
*B. longum* subsp. *longum*	DLBL07	DSM25669	JAGGDA000000000	PFGE, FTIR, WGS, and MLST
*B. longum* subsp. *longum*	DLBL09	DSM25671	JAGGCZ000000000	PFGE, FTIR, WGS, and MLST
*B. animalis* subsp. *lactis*	BS01	LMG P-21384	JAGGCY000000000	PFGE, FTIR, WGS, and MLST
*B. animalis* subsp. *lactis*	BS05	DSM23032	JAGGCX000000000	PFGE, FTIR, WGS, and MLST
*B. animalis* subsp. *lactis*	MB2409	DSM23733	JAGGCW000000000	PFGE, FTIR, WGS, and MLST
*B. animalis* subsp. *lactis*	BB12	ATCC 27673	CP001853.1	PFGE, FTIR, WGS, MLST
*B. longum* subsp. *longum*	BL-CT	DSM20219^T^	FNRW00000000.1	PFGE, FTIR, WGS, and MLST
*B. animalis* subsp. *lactis*	BS-CT	DSM10140^T^	CP001601.1	PFGE, FTIR, WGS, and MLST

*FTIR, Fourier transform infrared; MLST, multilocus sequence typing; PFGE, pulsed-field gel electrophoresis; WGS, whole-genome sequencing.*

**TABLE 2:** Additional type strains of *Bifidobacterium* included in the analyses.

**Species**	**Code**	**Deposit code**	**Techniques used**
*B. bifidum*	BB-CT	DSM20456^ T^	PFGE, FTIR
*B. breve*	BR-CT	DSM20213^ T^	PFGE, FTIR
*B. adolescentis*	BA-CT	DSM20083^ T^	PFGE, FTIR
*B. longum* subsp. *infantis*	BI-CT	DSM 20088^ T^	PFGE, FTIR

*FTIR, Fourier transform infrared; PFGE, pulsed-field gel electrophoresis.*

The strains were maintained at −80°C and subcultured in MRS broth (Difco) anaerobically at 37°C for 72 h.

### Pulsed-Field Gel Electrophoresis (PFGE) Analysis

The protocol was described by Tynkkynen et al. (1999). Cell suspension was mixed with an equal volume of 2% agarose gel (Pulsed Field Certified Agarose; Bio-Rad) prepared in 0.125 M ethylenediaminetetraacetic acid (EDTA) (pH 7.6) and dispensed into disposable plug molds (10 mm × 5 mm × 1.5 mm; Bio-Rad). The plugs were incubated in 1 ml of 1 M NaCl, 6 mM Tris–HCl, 100 mM EDTA, 1% Sarkosyl buffer (pH 7.6; Sigma) with 10 mg/ml lysozyme (Sigma), and 500 units/ml mutanolysin (Promega) at 37°C for 18 h. The plugs were then incubated in fresh Sarkosyl buffer with 1 mg/ml proteinase K (Sigma) at 37°C for 48 h.

The plugs were washed twice with 1 mM phenylmethylsulfonyl fluoride (PMSF; Sigma) in 10 mM Tris–HCl, 1 mM EDTA (pH 8.0) at 37°C for 60 min in a shaking water bath. Two slices (2-mm wide) were prepared from the plugs and washed three times in 1 ml of 10 mM Tris–HCl, 0.1 mM EDTA (pH 8.0) for 60 min at room temperature (r.t.). The slices were preincubated at r.t. for 30 min in 500 μl of the appropriate restriction endonuclease buffer. They were then transferred to 500 μl of a fresh restriction digest mixture containing 40 units of *Xba*I and incubated at 37°C for 18 h.

Electrophoresis (Briczinski and Roberts, 2006) was performed on 1.0% agarose gel (Bio-Rad) using 0.5 × TBE buffer (45 mM Tris, 45 mM boric acid, 1 mM EDTA, pH 8.0). A lambda PFG ladder (BioLabs N.E.) was included as a molecular weight marker. Electrophoresis was performed using a CHEF III System (Bio-Rad). Switch times were increased linearly from 0.2 to 35.4 s for 14 h, with an angle of 120° at 6 V/cm and 14°C. Gels were stained with a solution of ethidium bromide (0.4 mg/L; Promega) for 1 h, then destained for 30 min. Restriction patterns were visualized on a UV transilluminator, and images were captured using a GelDocXR System (Bio Rad) and saved as TIFF files for future analysis.

### Whole-Genome Sequencing (WGS) and OrthoANI Calculation

Total DNA was extracted from the strains using the Wizard genomic DNA purification kit (Promega, Mannheim, Germany) according to the manufacturer’s recommendations. The concentration of the DNA was measured by a Nanodrop Lite spectrophotometer (Thermo Scientific, Waltham, MA, United States) and a Qubit 4.0 fluorometer (Life Technologies; Invitrogen, CA, United States). Furthermore, the quality of the DNA was assessed on agarose gel electrophoresis. The DNA was stored at −20°C prior to WGS.

The WGS was performed for the strains reported in Table 1, except for the strains *B. longum* subsp. *longum* W11, *B. animalis* subsp. *lactis* BB12, and the type strains of both species, whose genome sequences were available at the time of the study in GenBank database (Table 1). Genome sequences were determined using Illumina MiSeq sequencing technology with a 300-paired-end library. The sequences of adapters were searched and removed from the reads using cutadapt tool (Martin, 2011) and applying BLASTn searches with a minimum evalue 1e-05. Subsequently, quality trimming was performed on the reads with Erne-filter (Del Fabbro et al., 2013) using default parameters, and only sequences passing the quality thresholds were assembled into contigs by CLC Workbench v7. The genome sequences thus obtained were deposited at DDBJ/ENA/GenBank under the accession reported in Table 1.

The whole genome similarity between the strains of the species *B. longum* subsp. *longum* and *B. animalis* subsp. *lactis* was determined through the comparison of the OrthoANI values calculated by means of the OAT software (Lee et al., 2016).

### Multilocus Sequence Typing (MLST) Analysis

The genome sequences of the strains reported in Table 1 were used to retrieve the nucleotide sequence of the seven loci included in the MLST scheme described by Delétoile et al. (2010). The MLST sequences obtained by BLASTn searches were used for the construction of phylogenetic tree based on the concatenated sequence of the genes *clpC*, *fusA*, *gyrB*, *ileS*, *purF*, *rplB*, and *rpoB*. Multiple alignments were made through ClustalX 2.1, and the tree was reconstructed using the neighbor-joining method and Jukes–Cantor substitution model.

### Fourier Transform Infrared (FTIR) Analysis

An IR Biotyper spectrometer (Bruker Optics-Daltonics GmbH) was used for this investigation.

An amount of 1 μl overloaded loop of bacterial colonies taken from the confluent part of the culture were resuspended in 50 μl of 70% ethanol solution in an IR Biotyper suspension vial. After vortexing, 50 μl of deionized water were added, and the solution was mixed by pipetting. Here, 15 μl of the bacterial suspension were spotted in four technical replicates onto the 96-spot silicon IR Biotyper target and let dry for 15–20 min at 35°C ± 2°C.

In each run, prior to sample spectra acquisition, quality control was performed with the Infrared Test Standards (IRTS 1 and 2) of the IR Biotyper kit. IRTS 1 and IRTS 2 were resuspended in 90 μl of deionized water; 90 μl of absolute ethanol were added and mixed. Here, 12 μl of suspension were spotted in duplicate onto the IR Biotyper target and let dry as previously described for the samples. After spectra acquisition and evaluation of IRBT1 and IRBT2, spectra of the samples were acquired, intercalating a background spectrum between each sample.

Spectra acquisition, visualization, and processing were performed in transmission mode in the spectral range 4,000–600 cm^–1^ (mid-IR). The second derivative of the spectra was calculated using Savitzky–Golay algorithm over nine data points. Spectra were then cut to 1,300–800 cm^–1^ (Van der Mei et al., 1993) and vector-normalized to amplify differences between isolates, and correct variations related to spectra acquisition.

First, the discriminatory power of IR Biotyper was investigated within the *Bifidobacterium* genus using six different *Bifidobacterium* species (*B. animalis* subsp. *lactis*, *B. longum* subsp. longum, *B. longum* subsp. *infantis*, *Bifidobacterium bifidum*, *Bifidobacterium breve*, and *Bifidobacterium adolescentis*) (Table 2). After that, the discriminatory power within the *B. animalis* subsp. *lactis* and *B. longum* subsp. *longum* species was evaluated (Table 1). Hierarchical cluster analysis (HCA) to assess the similarity of the samples was performed by means of the IR Biotyper Client software v1.5 using Euclidean metric and single linkage. For each dataset explored, the IR Biotyper software automatically calculated a clustering cutoff, which is the product of the Simpson’s index of diversity and the mean coherence of the parameter defined by the user (individual strain, in this study) (Hunter and Gaston, 1988). For each dataset, a strain belonging to another species was included to have a reference of “non-relatedness.”

## Results

### *Bifidobacterium longum* subsp. *longum*

**FIGURE 1:**
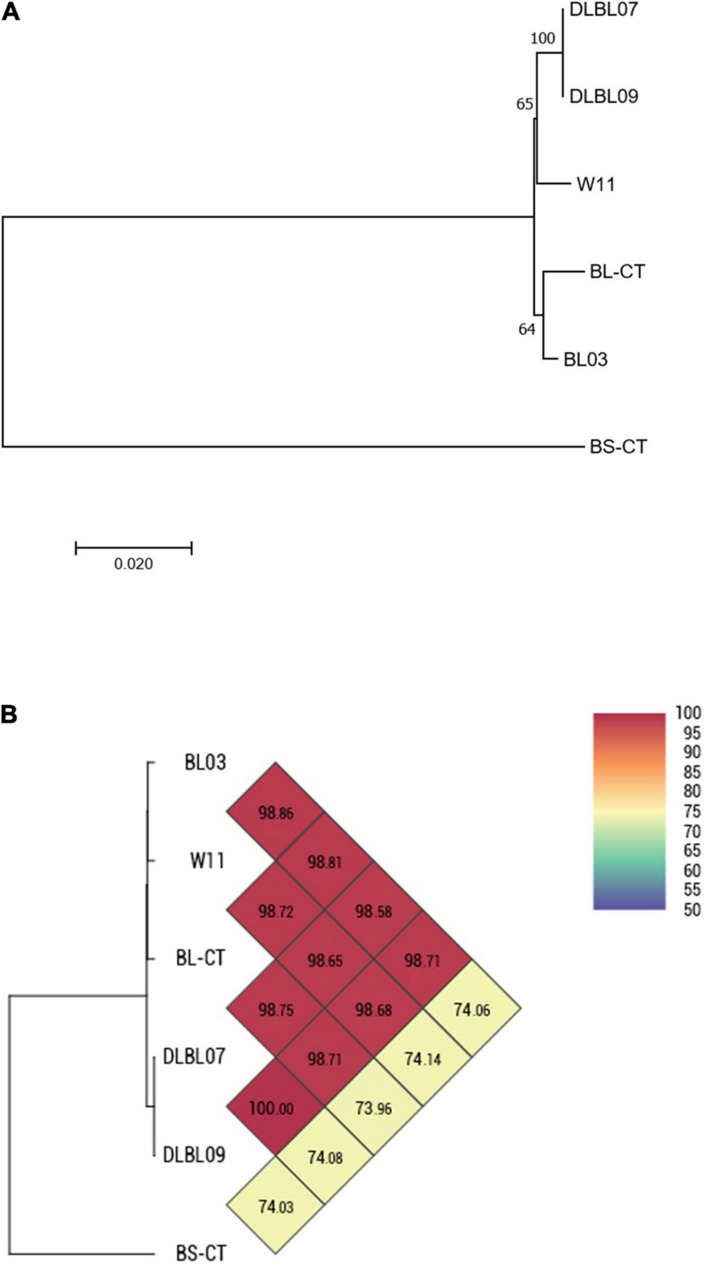
Whole-genome comparison for *Bifidobacterium longum* subsp. *longum* strains based on the multilocus sequence typing (MLST) analysis and the OrthoANI values. **(A)** Phylogenetic tree based on the concatenated nucleotide sequences of the genes *clpC*, *fusA*, *gyrB*, *ileS*, *purF*, *rplB*, and *rpoB* included in the MLST scheme for *B. longum* subsp. *longum* strains. Bootstrap values (1,000 replicates) are shown as a percentage at the branching points. The scale bar represents the number of nucleotide substitutions per site. **(B)** Heatmap generated with OrthoANI values calculated from the OAT software for *B. longum* subsp. *longum* strains.

Four strains of *B. longum* subsp. *longum* (BL03, W11, DLBL07, and DLBL09) and a type strain (BL-CT) were examined. The MLST analysis confirmed that the strains belong to the species *B. longum* subsp. *longum*, clustering separately from the type strain *B. animalis* subsp. *lactis* BS-CT, used as reference control (Figure 1A). The MLST analysis revealed a very high relatedness between the strain DLBL07 and the strain DLBL09, which constituted a single phylogenetic cluster, while the strains W11 and BL03 and the type strain BL-CT were located in separated clusters (Figure 1A). Similarly to what was observed for the MLST analysis, strains DLBL07 and DLBL09 shared an OrthoANI value of 100%, while they exhibited values ranging from 98.58 to 98.71% when they were compared with the strains W11 and BL03 (Figure 1B). The comparison between the analyzed strains and the type strain BL-CT displayed OrthoANI values between 98.68 and 98.86%.

**FIGURE 2:**
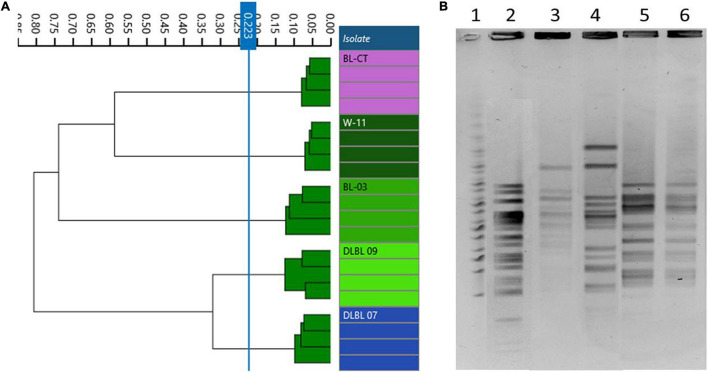
Comparison of Fourier transform infrared (FTIR) and pulsed-field gel electrophoresis (PFGE) typing methods on *B. longum* subsp. *longum* strains. **(A)** Dendrogram obtained by clustering FTIR spectra for *B. longum* subsp. *longum* strains (cut-off, 0.223). **(B)** *B. longum* subsp. *longum* strain PFGE profiles with *Xba*I (1) Marker; (2) BL-CT; (3) BL03; (4) W11; (5) DLBL07; (6) DLBL09.

In accordance with genomic results, different PFGE patterns were evidenced for the strains W11 and BL03 and the type strain BL-CT, while strains DLBL07 and DLBL09 showed the same PFGE profile and were not discernible (Figure 2B).

Dendrograms built from FTIR spectra acquisition evidenced five different types corresponding to the five strains used in this study (cutoff, 0.223); in particular, FTIR spectroscopy was able to distinguish correctly the strains DLBL07 and DLBL09 (Figure 2A).

### *Bifidobacterium animalis* subsp. *lactis*

**FIGURE 3:**
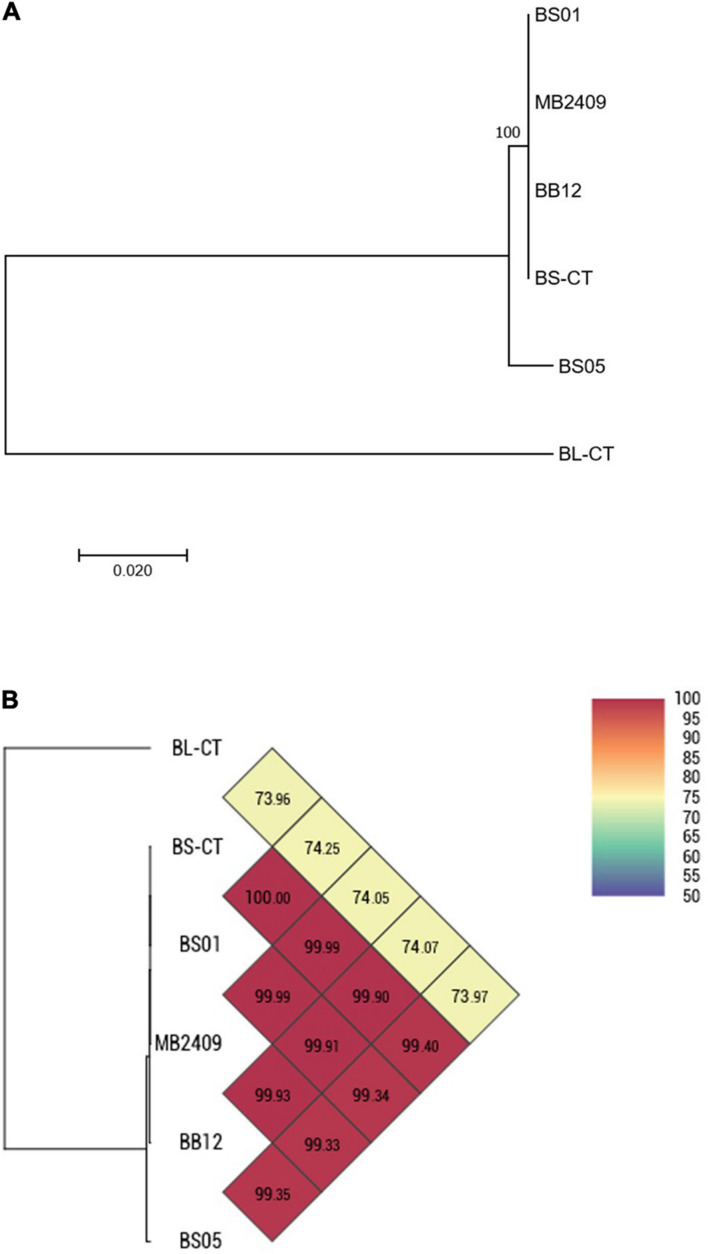
Whole-genome comparison for *B. animalis* subsp. *lactis* strains based on the multilocus sequence typing (MLST) analysis and the OrthoANI values. **(A)** Phylogenetic tree based on the concatenated nucleotide sequences of the genes *clpC*, *fusA*, *gyrB*, *ileS*, *purF*, *rplB*, and *rpoB* included in the MLST scheme for *B. animalis* subsp. *lactis* strains. Bootstrap values (1,000 replicates) are shown as a percentage at the branching points. The scale bar represents the number of nucleotide substitutions per site. **(B)** Heatmap generated with OrthoANI values calculated from the OAT software for *B. animalis* subsp. *lactis* strains.

Four strains of *B. animalis* subsp. *lactis* (BS01, BS05, MB2409, and BB12) and a type strain (BS-CT) were processed. The MLST analysis confirmed that the strains belong to the species *B. animalis* subsp. *lactis*, clustering separately from the type strain *B. longum* subsp. *longum* BL-CT, used as reference control (Figure 3A). However, only BS05 was easily distinguished, while strains BS01 and MB2409 constituted a separate single phylogenetic cluster with strain BS-CT and probiotic strain BB12, as reported in Figure 3A. The high similarity between strain BS01 and type strain BS-CT was confirmed by the shared OrthoANI value that corresponds to 100% (Figure 3B). Moreover, within the strains examined for the species *B. animalis* subsp. *lactis*, BS05 exhibited the lowest OrthoANI values compared to the strains under analysis, which are between 99.33 and 99.40% (Figure 3B).

**FIGURE 4:**
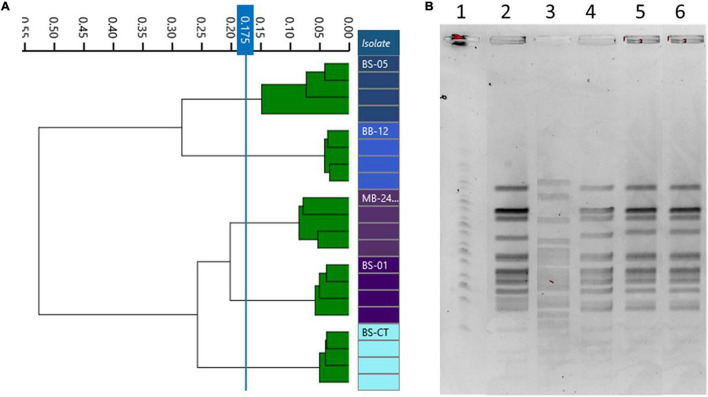
Comparison of Fourier transform infrared (FTIR) and pulsed-field gel electrophoresis (PFGE) typing methods on *B. animalis* subsp. *lactis* strains. **(A)** Dendrogram obtained by clustering FTIR spectra for *Bifidobacterium animalis* subsp. *lactis* strains (cut-off, 0.175). **(B)** *B. animalis* subsp. *lactis* strain PFGE profiles with *Xba*I (1) Marker; (2) BS01; (3) BS05; (4) MB2409; (5) BB12; (6) BS-CT.

The same homogeneous trend was observed in PFGE patterns. In fact, BS05 was the only strain showing a discernible profile, while BS01, MB2409, and BB12 exhibited the same PFGE pattern (Figure 4B). Moreover, the type strain BS-CT shared the same PFGE profile of BS01, MB2409, and BB12.

Differently, dendrograms built from FTIR spectra acquisition classified the examined isolates in different clusters, distinguishing all the strains used as different types, corresponding to BS01, BS05, MB2409, and BB12 and type strain BS-CT (cutoff, 0.173) (Figure 4A).

### *Bifidobacterium* Species/Subspecies

**FIGURE 5:**
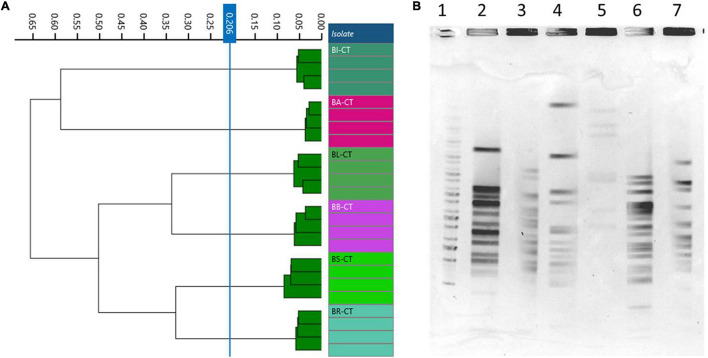
Comparison of Fourier transform infrared (FTIR) and pulsed-field gel electrophoresis (PFGE) typing methods on *Bifidobacterium* species/subspecies. **(A)** Dendrogram obtained by clustering FTIR spectra for *Bifidobacterium* type strains (cut-off, 0.206). **(B)** Type strain PFGE profiles with *Xba*I (1) Marker; (2) BA-CT; (3) BI-CT; (4) BR-CT; (5) BB-CT; (6) BL-CT; (7) BS-CT.

A total of six species or subspecies belonging to *Bifidobacterium* genus, BA-CT, BI-CT, BR-CT, BB-CT, BL-CT, and BS-CT, were subjected to PFGE and FTIR analysis in order to evaluate the discriminatory power of FTIR spectroscopy also at the species/subspecies level. Moreover, a comparison between FTIR and PFGE has been carried out. No contradictory results were evidenced from the two techniques: PFGE showed six different profiles, and FTIR distributed the isolates in six clusters, corresponding to the species/subspecies tested (cutoff, 0.206) (Figures 5A,B).

## Discussion

Microorganisms are known to play a crucial role in maintaining the human health status: some bacteria, invading the host, can cause a range of diseases, but several others, establishing a mutualistic relationship with the human body, contribute to the normal host physiology. The administration of beneficial microorganisms may represent a key determinant of the general health status and disease susceptibility. Therefore, bacterial typing at the strain level represents a great challenge to human health, considering not only factors associated with harmful microorganisms, such as increased virulence, transmissibility of pathogens, resistance to multiple antibiotics, but also the favorable side, including the mechanisms that lead to benefits deriving from probiotics administration (Fournier et al., 2004).

The discriminatory power of FTIR spectroscopy by typing at the species and strain level bacteria belonging to *Bifidobacterium* genus was assessed in this study. The procedure was successful in distinguishing the different species/subspecies tested (*B. bifidum*, *B. breve*, *B. adolescentis*, *B. longum* subsp. *infantis*, *B. longum* subsp. *longum*, and *B. animalis* subsp. *lactis*) and, surprisingly, also the different strains belonging to *B. longum* subsp. *longum* and *B. animalis* subsp. *lactis*. Although the functionality of FTIR method in species typing has been already verified for Gram-negative bacteria, such as *E. coli*, *Klebsiella oxytoca*, and *Y. enterocolitica* (Kuhm et al., 2009; Sousa et al., 2013; Dieckmann et al., 2016), and isolates responsible for hospital outbreaks, such as *Enterobacter cloacae*, *K. pneumoniae*, and *Pseudomonas aeruginosa* (Martak et al., 2019; Hu et al., 2020), its discriminatory power has not been assessed for Gram-positive and probiotic bacteria until now.

Fourier transform infrared provided potentially equivalent data with respect to those resulting from the gold standard PFGE and MLST, which discriminated the strains belonging to the *B. longum* subsp. *longum* group, with the exception of DLBL07 and DLBL09, and the strain BS05 of the *B. animalis* subsp. *lactis* group. Surprisingly, FTIR technology was able to discriminate DLBL07 and DLBL09, which, on the contrary, clustered together in MLST and PFGE analyses, and therefore they were not discernible with these DNA-based methods. Another unexpected and relevant result was that FTIR revealed to be more informative for *B. animalis* subsp. *lactis* group with respect to the other two techniques, distinguishing all the strains belonging to this group, while MLST and PFGE did not reach this result, but they were only able to discriminate BS05. Moreover, the typing power of FTIR within the *Bifidobacterium* genus was demonstrated both at the species level and at the strain level.

It is important to consider that while gold standard methods that have been tested in this study are DNA-based techniques, FTIR technology acquires spectra deriving from the carbohydrate composition of the bacterial cell wall. Consequently, MLST and PFGE detect genomic variations that may lead to alterations in carbohydrate composition, but FTIR spectroscopy can highlight modifications in bacteria wall that probably are not always attributable to genetic differences.

*Bifidobacterium longum* subsp. *longum*, classified as subspecies together with *B. longum* subsp. *infantis* and *B. longum* subsp. *suis* of the species *B. longum* (Mattarelli et al., 2008), is considered one of the most important contributors to host health, and representative strains are frequently used as probiotics, fermented products, and pharmaceutical preparations. *B. longum* strains have been demonstrated to have a high genomic heterogeneity in PFGE analysis (Roy et al., 1996; Ventura et al., 2004; Ward and Roy, 2005), in accordance with our results.

*Bifidobacterium animalis* subsp. *lactis*, described by Masco et al. (2004) as subspecies belonging to the species *B. animalis*, is currently the most utilized probiotic species among the bifidobacteria (Mattarelli and Biavati, 2017). However, from a genetic point of view, this subspecies, exhibiting a huge sequence similarity among the different strains presently described, has been named “monomorphic” or “monophyletic” (Milani et al., 2013). It is possible that the large importance acquired in the probiotic industry led to a lack of diversity within *B. animalis* subsp. *lactis* strains. In fact, the intense focus on commercially relevant strains could have resulted in a reisolation of the same strains and assignment as new ones. Loquasto et al. (2013) supported this hypothesis demonstrating that *B. animalis* subsp. *lactis* ATCC 27673, isolated from sewage, constituted a genetically distinct strain with respect to other strains of *B. animalis* subsp. *lactis* isolated from human feces. Despite the lack of genetic variability, strains of *B. animalis* subsp. *lactis* have been shown to differ in phenotypic characteristics, such as in resistance to oxidative stress (Oberg et al., 2011). Therefore, FTIR spectroscopy, according to our results, which demonstrated the highest discriminatory power with respect to PFGE and MLST on *B. animalis* subsp. *lactis* strains, can overcome genetic limitations regarding this monomorphic subspecies, evidencing potential phenotypes that can be beneficial for industrial or human health purposes.

Probiotic products are unique in their properties to confer a health benefit, and they can present different challenges in design, development, scale-up, manufacturing, commercialization, and life cycle management (Jackson et al., 2019). Quality and safety assessment of probiotic food and supplements is a responsibility of the industry, and FTIR spectroscopy constitutes a method that can be inserted in the process for rapid biotyping of different strains belonging to the same species.

In the scenery of clinical application, it should be pointed out that matching the appropriate probiotic strain to patients who suffer from a certain disease can represent a challenging task. In this regard, McFarland et al. (2018) demonstrated the importance of considering both probiotic strain specificity and disease specificity. Based on this evidence, a technology that is able to guarantee a clear discrimination among probiotic strains can be considered essential for the design of clinical trials focused on the prevention or treatment of diseases. FTIR spectroscopy, with the potential highlighted from this study in discriminating bifidobacteria, can be successfully inserted in the process of the clinical choice of the suitable probiotic strain, accelerating and implementing the clinical strategy.

The *Bifidobacterium* genus has always gained high microbiological interest due to its potentially health-promoting effects and increasing use as a probiotic. The comparison of important characteristics of bifidobacterial species and strains, such as interactions with the host, gut colonization dynamics, or ecological distribution, is object of intensive studies in probiotic industries. As probiotic effects are species- and even strain-specific, the European Food Safety Authority requests a precise characterization of food constituents that are microorganisms, which are the subject of health claims (EFSA Panel on Dietetic Products, Nutrition and Allergies, 2009).

In the perspective of growing demand on probiotic bacteria, industries need rapid and accurate identification of specific bacteria. Moreover, isolation of new microorganisms from various environments may lead to multiple isolations of the same strain. Only a few studies focused on the use of FTIR and vibrational spectroscopic techniques to investigate lactic acid bacteria and probiotics (Wenning et al., 2010; Santos et al., 2015), including *Bifidobacterium* at species level (Mayer et al., 2003), but the investigation on bifidobacteria at the subspecies level has been unexplored until the current study. Therefore, it is important to apply rapid, low-cost, and effective procedures able to differentiate bifidobacteria strains. In this context, the correct identification of probiotic strains can provide a useful framework for examining the evolutionary dynamics and phylogenetic distribution of significant strain properties. PFGE being quite laborious and MLST considerably expensive, and both time-consuming (2–3 days), particularly when a large group of new isolates is typed, their routine application for probiotic identification can be demanding. Instead, FTIR spectroscopy, as a quick, inexpensive, and high-throughput tool for bacterial typing, provides reliable discriminatory information, based on our results regarding bifidobacteria, and with the availability of well-composed databases, it can constitute a suitable and appropriate method for typing bifidobacteria in the frame of probiotic production. Although our investigation was limited to a restricted number of strains belonging to *Bifidobacterium*, the results obtained are extremely promising, supporting the application of this method also to other probiotic cultures.

## Conclusion

We demonstrated that FTIR spectroscopy successfully discriminated a group of bacteria traditionally used as probiotics, bifidobacteria, at the species/subspecies and strain levels. The typing functionality was not only equivalent compared to two other consolidated techniques but also more informative especially for *B. animalis* subsp. *lactis* strains. In addition, FTIR technology can be suitable for the routine of probiotic industry laboratories—thanks to its huge advantages with respect to DNA- based techniques, such as the ease of use, the fast turnaround time, the user-friendly software, and the relatively low running costs. This study can pave the way for the use of FTIR in the probiotic industry for typing other species belonging to *Bifidobacterium*, other genera/species traditionally used as probiotics, and novel strains with probiotic potential.

## Data Availability Statement

The dataset including the genome sequence of the strain BL03, DLBL07, DLBL09, BS01, BS05, and MB2409 analyzed in this study can be found in the BioProject PRJNA716495 at DDBJ/ENA/GenBank database, where the genome sequence are available under the accession JAGGDB000000000, JAGGDA000000000, JAGGCZ000000000, JAGGCY000000000, JAGGCX000000000, and JAGGCW000000000, respectively. The version described in this paper is the first version.

## Author Contributions

MP and FD conceived the study. FD performed the PFGE analysis. MC carried out the FTIR experiments. FF and IC performed the MLST and OrthoANI analyses. NBC interpreted the results and contributed to the writing of the manuscript. DDG, MP, and SA critically revised the final manuscript. All authors contributed to the article and approved the submitted version.

## Conflict of Interest

MP and FD are employees of Probiotical Research S.r.L., Novara, Italy. MC is employee of Bruker Daltonik, Bremen, Germany. FF and IC are employees of Microbion S.r.L., San Giovanni Lupatoto, Verona, Italy. The remaining authors declare that the research was conducted in the absence of any commercial or financial relationships that could be construed as a potential conflict of interest.

## Publisher’s Note

All claims expressed in this article are solely those of the authors and do not necessarily represent those of their affiliated organizations, or those of the publisher, the editors and the reviewers. Any product that may be evaluated in this article, or claim that may be made by its manufacturer, is not guaranteed or endorsed by the publisher.
